# The effect of green mussel ( *Perna viridis*) shells’ hydroxyapatite application on alkaline phosphatase levels in rabbit femur bone defect

**DOI:** 10.12688/f1000research.132881.2

**Published:** 2024-06-07

**Authors:** Kevin Christian Tjandra, Robin Novriansyah, Edward Kurnia Setiawan Limijadi, Lydia Kuntjoro, Meita Hendrianingtyas

**Affiliations:** 1 Kariadi General Hospital, Semarang, Indonesia; 2 Department of Medicine, Faculty of Medicine, Universitas Diponegoro, Semarang, Central Java, Indonesia; 3 Department of Surgery, Faculty of Medicine, Universitas Diponegoro, Semarang, Central Java, Indonesia; 4 Department of Clinical Pathology, Faculty of Medicine, Universitas Diponegoro, Semarang, Central Java, Indonesia; 5 Department of Radiology, Medical Faculty, Universitas Diponegoro, Semarang, Central Java, Indonesia

**Keywords:** Hydroxyapatite, green mussel shell, bone substitute, bone healing, alkaline phosphatase

## Abstract

**Background:**

Non-union fractures can be prevented with bone grafts, such as hydroxyapatite made from green mussel shells. Green mussel shells contain a high percentage of HA, making them a promising alternative for bone healing. This research aims to reveal the effectiveness of green mussel shell HA as a bone substitute material and to provide knowledge for further research.

**Methods:**

This research was conducted for four months using a true experimental research method with a post-test-only control group design. This study used 36 New Zealand rabbits ( *Oryctolagus cuniculus*) which were divided into 9 groups: positive control, negative control, and intervention at weeks 2, 4 and 6 after the intervention. All groups were subjected to three general procedures: pre-surgery, surgery, and post-surgery. This study utilized histological evaluation and biochemical assessment, specifically measuring serum alkaline phosphatase (ALP) levels, to investigate the effects of hydroxyapatite (HA) from green mussel shells on bone healing in rabbits.

**Results:**

The findings demonstrated that green mussel shell HA hashad efficacy in accelerating bone healing, better than HA bovine HA i.e. green mussel shell hydroxyapatite showed superior efficacy compared to bovine hydroxyapatite in accelerating and maximizing fracture healing, as compared to the 6-week negative control group and demonstrated a significant difference ( *p* < 0.05).

**Conclusions:**

Green mussel hydroxyapatite is proven to be able to fasten and maximize the bone healing process as fast as bovine HA, and even has higher efficacy than bovine HA.

## Introduction

The incidence of traffic accidents has increased in Indonesia, which often cause disability in the form of fractures. ^1^^,^^2^ Based on data recorded by the Directorate of Traffic of the Regional Police of Central Java in 2018, the prevalence of fractures due to traffic injury is 5.5% in Indonesia. ^3^ The Global Burden of Diseases, Injuries, and Risk Factors Study (GBD) 2019 reported by the GBD 2019 Fracture Collaborators provides information on the global burden of fractures. In 2019, there were 178 million (95% uncertainly interval [UI] 162–196) new fractures, 445 million (428–484) prevalent fractures, and 25·8 million (17·8–35·8) years lived with disability (YLDs) due to fractures. ^4^ A study found that patients experienced a significant impairment in health-related quality of life (HRQOL) after a hip fracture, particularly in self-care, pain/discomfort, and usual activities. ^5^ The study also noted that fear of falling and lack of confidence were common among patients after hip fracture, which could contribute to functional decline. If these conditions are not handled, the decline in quality of life and activity limitations cannot be avoided.

Fracture is a condition where there is a discontinuity of bone. ^6^ Meanwhile, bones have the ability to heal. ^1^ Fracture healing necessitates a combination of mechanical stability from appropriate fixation, adequate bone vascularization, osteoprogenitors and growth factors from bone cells, and interaction between shattered bone fragments. Non-union fractures can occur if a combination of these conditions is not met. ^7^^,^^8^ As a result, bone grafts with osteogenesis, osteoinduction, and osteoconduction characteristics are required to assist in the healing of acute fractures and non-union fractures. ^9^

A material called hydroxyapatite (HA) is frequently utilized in the development of bone substitutes. This is because HA makes up 50% of the mineral components of bone. Bone consists of 69% mineral components, 22% organic matrix, and 9% water. HA is a key component needed in the process of bone regeneration and healing. ^10^ Virgin clam shells and green mussel shells are two examples of wastes that could serve as a source of HA for bone substitutes. ^11^^,^^12^ The virgin clam shell ( *Anadara granosa*) has been suggested as a viable material for the synthesis of HA. ^13^ On the other hand, no recent studies related to green mussel shells have been conducted. Therefore, the potential of green mussel shells as a material for HA synthesis will be examined in this research. ^14^

Green mussel shells contain two polymorphs of calcium carbonate (CaCO _3_), namely calcite and aragonite. ^15^ Most organic compounds can be found among the crystallites (intercrystalline), but some organic molecules (intercrystalline) are also intercalated in the crystal lattice. More specifically, green mussel shells consist of 95-99% CaCO _3_ (calcite, aragonite, or vaterite) with lesser amounts of MgCO _3_, Al (Fe _2_O _3_), SiO _2_, Ca _3_P _2_O _8_, CaSO _4_, proteins, and mucopolysaccharides. This CaCO _3_ content will then be processed into the hydroxyapatite. ^16^^,^^17^

Moreover, the crystalline structure of virgin clam shells and green mussel shells fosters HA formation, featuring two polymorphs of calcium carbonate: calcite and aragonite. ^18^ By processing these shells, the calcite and aragonite forms of calcium carbonate can be converted into HA, enhancing its bioactivity and compatibility. Compared to other sources of HA like bone or coral, clam and mussel shells have several advantages. ^19^ They are abundant and can regenerate quickly, making them sustainable and reducing environmental impact. Additionally, their composition closely resembles human bone, minimizing the risk of negative reactions. Therefore, utilizing clam and mussel shells as sources of HA has great potential for efficient and sustainable bone regeneration therapies in the field of biomaterials and tissue engineering.

Indonesia is able to produce 140 – 210 tons per hectare of green mussel shell waste every year. ^20^ Green mussel shells consist of 95.69% HA, so 133.97 – 287.07 tons per hectare of HA can be produced annually. ^20^ Therefore, green mussel shells have the potential to be an alternative material in the production of HA.

Alkaline phosphatase (ALP), an enzyme that contributes to the process of bone mineralization, is produced by osteoblasts with increased activity. As one of the elements of a complete blood count, ALP can therefore serve as a biomarker that is frequently evaluated and easily accessed to detect bone healing. ^21^ Alkaline phosphatase (ALP) is an enzyme produced by osteoblasts that plays a crucial role in the process of bone mineralization. Elevated ALP levels are generally indicative of increased osteoblastic activity, which is essential for bone formation and repair. This correlation has been extensively studied in both human and animal models. In humans, studies have shown that ALP levels peak during the early stages of bone healing and gradually decrease as the bone matures. For instance, Rathwa et al. noted that peak serum ALP levels in fracture patients occurred around the sixth week, aligning with the period of active bone formation. Elevated ALP levels are observed during the initial stages of bone healing, reflecting increased osteoblastic activity. In this study, serum ALP levels were measured at weeks 2, 4, and 6 to assess the impact of green mussel shell hydroxyapatite (HA) on bone healing. The results indicated that while ALP activity varied at different time points, the differences were not statistically significant, suggesting that the effect of HA was consistent throughout the healing process. Overall, the positive correlation between ALP serum levels and bone healing processes is supported by both human and animal studies, making ALP a reliable biomarker for assessing bone healing efficacy.

In this research, serum ALP levels will be measured in the weeks 2, 4, and 6 to see how the distribution of green mussel shell HA affects the ALP levels. This retrieval was timed in accordance with findings from Rathwa et al., ^22^ who noted that peak serum ALP levels in fracture patients occurred in the sixth week. ^22^ Alkaline phosphatase (ALP) is a vital enzyme that plays a significant role in the process of bone mineralization. Typically, ALP levels increase during the early stages of bone healing as osteoblasts, cells responsible for bone formation, become more active. According to Rathwa et al., the peak serum ALP levels in patients with fractures usually occur around the sixth week after the injury. This peak indicates a period of maximum osteoblastic activity and bone formation.

This research aims to observe the effectiveness of green mussel shell HA as a bone substitute material and to provide knowledge for further research. The primary objective of this study is to investigate the impact of hydroxyapatite (HA) derived from green mussel (Perna viridis) shells on alkaline phosphatase (ALP) levels in rabbit femur bone defects. Specifically, the study aims to evaluate the efficacy of green mussel shell HA in accelerating bone healing compared to traditional bovine HA. By assessing the ALP levels at different time points post-intervention, the study seeks to elucidate the potential of green mussel shell HA as a bone substitute material.

This research contributes to the field by introducing a novel approach to bone regeneration utilizing HA derived from green mussel shells. While HA is a well-known component in bone substitutes, the utilization of green mussel shells as a sustainable and abundant source of HA presents a unique avenue for exploration. Unlike previous studies that primarily focus on synthetic or bovine-derived HA, this study highlights the feasibility and efficacy of utilizing natural resources such as green mussel shells for HA synthesis. Furthermore, the study addresses a crucial gap in the existing literature by investigating the specific effects of green mussel shell HA on ALP levels in bone healing.

## Methods

### Ethics

Ethical clearance was issued by the Medical and Health Research Ethics Commission Faculty of Medicine, University of Diponegoro ( *Komite Etik Penelitian Kesehatan Fakultas Kedokteran Universitas Diponegoro*), with serial number 03/EC/H/FK-UNDIP/I/2022 approved on January 11 ^th^ 2021. All methods and protocol, including the research question, key design features, and analysis plan, were performed in accordance with the relevant guidelines and regulations and the study is reported in accordance with ARRIVE guidelines. All efforts were made to ameliorate any suffering of animals. All the efforts aim for acclimatization to account for their diverse origins; each rabbit was kept separately in polycarbonate cages (0.90 0.60 0.60 m) for a week on a 12-hour light/dark cycle at a constant temperature of 25°C and humidity of 50%. Animals were routinely observed for food consumption and fecal characteristics while being fed a conventional pellet diet and drinking tap water at will.

### Design

This research was carried out at the Animal Test Laboratory in the Lembah Kalipancur Tourism Village, Semarang, and the Healthy Animal Clinic Laboratory in Malang. This research was carried out for four months from September to December 2021. This study used a true experimental study with a post-test-only control group design.

### Animals and materials

The experimental animals used in this study were New Zealand guinea rabbits ( *Oryctolagus cuniculus*) obtained from rabbit breeders in Ambarawa, Semarang Regency. The inclusion criteria in this study were skeletally matured, male *O. cuniculus*, aged 6-12 months, with a weight of 2.5 – 3 kg. The exclusion criteria in this study were there being anatomical abnormalities, signs of infection, and rabbits that died during treatment. The required sample size was calculated using a resource equation. The equation showed that each group (9 groups) should consist of 3 rabbits with 1 additional rabbit to account for drop out (10%). Thus, 36 rabbits were used in this study.

**Figure 1.**
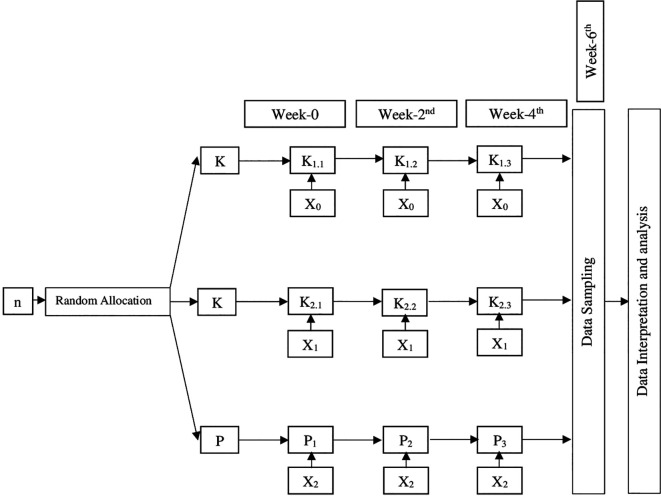
Study group design. Description: n: Sample K _1_: Negative control K _1.1_: Negative control observed sixth week after intervention K _1.2_: Negative control observed fourth week after intervention K _1.3_: Negative control observed second week after intervention K _2_: Positive control K _2.1_: Positive control observed sixth week after intervention K _2.2_: Positive control observed fourth week after intervention K _2.3_: Positive control observed second week after intervention P: Intervention group P _1_: Intervention group observed sixth week after intervention P _2_: Intervention group observed fourth week after intervention P _3_: Intervention group observed second week after intervention X _0_: No Intervention X _1_: Bovine HA implantation in rabbit femur X _2_: Green mussel shell HA implantation in rabbit femur

Sampling was conducted by simple random sampling to avoid bias due to variations in age and weight. Rabbits that met the inclusion and exclusion criteria were assigned randomly after being determined to be homogeneous using a simple randomization method. Then the 4 stages of blinding were conducted which included blinding during allocation, during the experiment, during the outcome assessment, and during the data analysis. After a week of adaptation, samples were obtained randomly from the group of *O. cuniculus.* Then the 36 rabbits were divided into 9 groups (4 rabbits for each group) as shown in Figure 1.

Bovine hydroxyapatite (HA) was selected as the positive control in this study due to various reasons. Firstly, HA derived from bovine sources is widely recognized and utilized in both research and clinical settings to enhance bone regeneration. By utilizing bovine HA as a positive control, the researchers were able to assess the efficacy of HA sourced from green mussel shells against established benchmarks in the field of medicine.

Seven rabbits from seven different groups (P _1_, K _1.1_, P _2_, K _2.2_, P _3_, K _2.3_, K _1.3_) were found dead during daily monitoring several days after the surgery with no specific cause of death, with the cause of death identified as infection and not specifically determined. None of the humane endpoint criteria ^23^ were noted prior to their death nor found on examination after death. Rabbits from P _1_ and K _1.1_ were found dead two days after the surgery, rabbits from P _2_ and K _2.2_ were found dead three days after the surgery, and rabbits from P _3_, K _2.3_, and K _1.3_ were found dead five days after the surgery. The final data does not include their information. However, the data is still credible since each group achieved the minimum sample of three rabbits for each of these groups.

### Procedure

The rabbits were selected according to the inclusion criteria after each rabbit was weighed. A total of 36 rabbits that met the inclusion criteria were then adapted, given food and drink as necessary for a week. During the adaptation period, rabbits were given vitamins A, D, E (0.1 mL/kg BW IM), vitamin B complex (0.1 mL/kg BW IM), and ivermectin (0.4 mg/kg BW IM) for disease prevention. After a week-long adaptation period, the rabbits were separated each into one cage according to their group. The rabbits were monitored for their humane endpoint conditions before and after the intervention for 2 hours per day (16:00 – 18:00). Those rabbits that reached their humane endpoint will be terminated by the administration of ≥100 mg/kg of pentobarbital (lethal dose) intravascularly (IV). The humane endpoint criteria can be seen in this table below, from Montgomery (1990). ^24^

The research data obtained were primary data from the observation of blood serum alkaline phosphatase levels as a bone healing biomarker on rabbit femur bones from the treatment group compared to the negative and positive control groups. The collection of blood serum alkaline phosphatase levels as biomarkers of bone healing was carried out in week 2, week 4, and week 6 after surgery.

The green mussel shell HA used in this study is obtained from the production led by Ismail *et al.* ^25^ at the Center of Biomechanics, Biomaterials, Biomechatronics, and Biosignal Processing (CBIOM3S). Then the obtained HA was powdered into a mortar and then the particle size was reduced using a stamper. This was done by creating a defect in the lateral part of the corpus femoris using a drill with a diameter of 5 mm and 500 rpm speed, then filling the defect with HA from green mussel shells. Pre-treatment of the defect site was done by ensuring minimal bleeding before applying hydroxyapatite (HA) powder. This step is important to prepare the area for more effective HA powder application. Next, the use of a biocompatible bonding agent is required to mix the HA powder, forming a paste or gel. This mixture will adhere more easily to the defect site and is resistant to displacement by blood. Finally, a coating technique is applied by adding HA powder in layers, allowing each layer to harden before adding the next. This approach helps to build a stable scaffold structure at the defect site.

In order to prevent infection, UV sterilization for ±3 hours were conducted. Before the implantation process, HA should be measured to prevent bias. UV sterilization is known to be effective in killing microorganisms on the surface of the material, which is crucial for ensuring that the implant surface is free of pathogens that can cause post-operative infections. In addition, this method has the advantage of having minimal influence on the physical and chemical properties of the implant material, so that the mechanical and biological properties of the material are maintained. This is in line with prvious study ^26^ on the ability of UV disinfection to reduce infection rates have been conducted. The required amount of HA is calculated by the following calculation:

m=ρ×V

$$m=3.18\;g/{\mathrm{cm}}^{3}\times 3.14\times 0.252\;\mathrm{cm}\times 0.5\;\mathrm{cm}$$

m=300mg

From the calculation, the mass of HA required for implantation of a tubular defect with a diameter of 5 mm and a depth of 5 mm is 300 mg.

There are two location options for making bone lesions in order to apply bone grafts, the metaphysis and the diaphysis. Both locations have advantages and disadvantages. There is a cushioning effect that prevents the risk of fracture due to the presence of layers of pars spongiosa and pars compacta which is an advantage of the metaphyseal section, but this section also has the disadvantage of being close to several large arteries and so there is risk of bleeding. In addition, the metaphysis has a lower volume and density than the diaphysis, making it easier to fracture. ^7^ The diaphysis location was chosen as the surgery site because it is far from the great artery and it is not easily fractured.

The surgical procedure began by shaving the hair at the incision area followed by injection of enrofloxacin 5 mg/kg IV in the rabbit’s ear through the *v. auricularis lateralis* as prophylactic antibiotics, and ketamine 10-40 mg/kg and acepromazine 3-5 mg/kg intramuscularly in *m. longissimus dorsi caudal* as the anesthetic. After that, an incision was made in the lateral area of the distal diaphysis of the *femur* with blade no. 22 by incising the fascia and periosteum of the rabbit femur according to the treatment group.

The procedure was conducted according to each of the experimental groups. K _1.1_, K _1.2_, K _1.3_: make a defect in the lateral body of the femoris using a drill with a diameter of 5 mm and a depth of 5 mm. The drilling equipment utilized originates from the Chinese manufacturer RUIJIN. Throughout the study, irrigation was implemented either continuously or intermittently while drilling to maintain a low temperature and cleanliness in the area. K _2.1_, K _2.2_, K _2.3_: make a defect in lateral corpus femoris using a drill with a diameter of 5 mm and a depth of 5 mm and fill the defect with bovine HA. P _1_, P _2_, P _3_: make a defect in the lateral part of the corpus femoris using a drill with a diameter of 5 mm and a depth of 5 mm and fill the defect with green mussel shell HA.

The fascia was sutured using absorbable catgut sutures, and the skin was sutured using non-absorbable silk. After that, the rabbits were examined until the time of data collection to know whether the rabbit showed any sign of infection, anatomical abnormalities, or died, so we could determine whether they met the exclusion criteria or not. The rabbits that showed exclusion criteria would be dropped out of the study.

The rabbits were taken care of properly by giving them food and drink as necessary until the time of blood serum data collection. The blood serum samples were taken two, four, and six weeks after surgery, depending on the intervention group of the rabbit. The intervention group is fully described in Figure 1. During this time, they were also given analgesic carbogen 2 mg/kg orally and antibiotic enrofloxacin 35mg/kg orally once per three days to keep their quality of life up.

The blood sampling was conducted by inserting needle parallel to the lateral marginal auricular vein. Once the needle reached the vein, blood will flow into the catheter. The vacutainer tube was pushed so that blood can enter the 1-2 ml tube. After collecting the required sample, the needle was immediately removed from the rabbit’s lateral marginal vein. The injected area was pressed with an alcohol swab at the end of this process. ^27^ HA implantation surgical technique is performed as follows:

1. The green mussel shell HA used in the study was obtained from the production led by Ismail et al at the Center of Biomechanics, Biomaterials, Biomechatronics, and Biosignal Processing (CBIOM3S).
2. The obtained HA was pulverized into powder using a pulverizer.
3. The particle size of HA was reduced using a stamper.
4. To prevent infection, UV sterilization is performed for about 3 hours.
5. Before the implantation process, HA is measured to prevent bias.
6. The required amount of HA is calculated based on certain calculations, such as the mass required for implantation of a tube defect with a diameter of 5 mm and a depth of 5 mm is 300 mg.

After the blood samples were collected, the rabbits were killed by the administration of ≥100 mg/kg of pentobarbital (lethal dose) intravascularly (IV). Then, they were monitored until a lack of heartbeat was noted for >60 seconds prior to carcass disposal.

Examination of ALP is selected due to its availability in most health centers. The availability of ALP screening in most health centers indicates that this test can generally be performed for a wide range of animal species, including rabbits. Detection of the ALP level from blood serum was carried out using the Mindray BC2800Vet series and blood chemistry analyzer with the brand Ubio-iChem-535 at the Animal Clinic Laboratory of Healthy Animals in Malang. In a previous study, this procedure was carried out based on measuring serum ALP levels in rabbit blood through the marginal vein. Then, the alkaline phosphatase is tested using 4-nitrophenol phosphate as a substrate and 2-amino-2-methyl-1-propanol (AMP) or diethanolamine (DEA) as a buffer, with DEA yielding higher ALP results. ALP isoenzymes can be differentiated using electrophoresis, HPLC, and other techniques. ^28^

### Analysis

The data obtained from the biochemical assessment were described by the ratio of serum alkaline phosphatase (ALP) levels. The type of ALP used in this study was EnzCheck Phosphatase Kit, Brand Bovine HA : Phaphros Indonesia.

The data analysis in the study was processed using SPSS version 25 software. The confidence interval (CI) used for the analysis was 95%. The technique used in sampling this study was simple random sampling. This technique was used to avoid bias due to variations in rabbit age and body weight. After meeting the inclusion and exclusion criteria, the rabbits were simply randomized to ensure homogeneity. The defect model in the study uses non-union fractures. A non-union fracture is a condition where broken bones fail to reunite after a sufficient period of time and adequate treatment. Criteria for a non-union fracture usually include the absence of signs of bone healing on radiographs after 6-9 months, persistent pain at the fracture site, and abnormal mobility at the fracture site.

The dose of hyaluronic acid (HA) administered in this study was based on previous studies and clinical guidelines. The specific dose used was 0.5 ml per injection site, which was administered weekly for a total of six weeks. Shapiro-Wilk test analyzed the normality of the data distribution. This test was chosen because the sample size in this study was <50. The data of this study were normally distributed with a p-value ≥0.05. The analysis was carried out using a one-way ANOVA test to see differences between groups because the data were normally distributed. A post hoc LSD test followed the results of the ANOVA test to find out more conclusively the relationship between variables that caused the data to be significant at the p-value <0.05. The comparison between the negative control group and the intervention group only shows a significant difference in the week 6.

In addition, data analysis was also carried out to compare the data for the week 2 and week 4; week 2 and week 6; and week 4 and week 6 using one-way ANOVA. The data of this study were considered insignificant at the p-value < 0.05.

## Results

In this study, a total of 36 New Zealand rabbits *O. cuniculus* were chosen and split into 9 groups. Each group consisted of four rabbits and were treated according to their group. The alkaline phosphatase blood serum level data was collected from the remaining 29 rabbits after 7 died before samples could be taken. The timing of data collection for rabbits was carried out according to the group, namely in the week 2, week 4, and week 6 after the intervention.

Table 1 shows the serum alkaline phosphatase (ALP) levels in rabbits in each treatment group. Differences in ALP levels were found vertically at weeks 2, 4, and 6. Horizontally, differences in ALP levels can be seen between the positive control, treatment, and negative control groups.

**Table 1.** Rabbit serum alkaline phosphatase level (U/L).

	Positive control ( *bovine*)	Negative control	Treatment (green mussel shell)
Week-2 ^nd^	44.33 (K _2.3_)	72.00 (K _1.3_)	60.50 (P _3_)
Week-4 ^th^	60.40 (K _2.2_)	57.70 (K _1.2_)	57.33 (P _2_)
Week-6 ^th^	42.17 (K _2.1_)	62.53 (K _1.1_)	18.65 (P _1_)

Table 2 shows that the results of the one-way ANOVA test, which reported a p-value = 0.483 and a Levene value = 0.244. Because the p-value was > 0.05, this analysis test showed no significant difference in serum ALP levels at week 2, week 4, and week 6 in the bovine group (K _2.3_, K _2.2_, and K _2.1_).

**Table 2.** One-way ANOVA test results for serum alkaline phosphatase levels in rabbits in the positive control group (bovine) (U/L).

Bovine	Mean ± SD	p	Levene
Week-2 ^nd^ (K _2.3_)	44.33 ± 19.00	0.483	0.244
Week-4 ^th^ (K _2.2_)	60.40 ± 7.24		
Week-6 ^th^ (K _2.1_)	42.17 ± 25.91		

Table 3 shows the results of the one-way ANOVA test which reported a p-value = 0.052 and a Levene value = 0.034. Because the p-value was > 0.05, this analysis test showed no significant difference in serum ALP levels at week 2, week 4, and week 6 of the green mussel shell group (P _3_, P _2_, and P _1_).

**Table 3.** One-way ANOVA test results for serum alkaline phosphatase levels in rabbits in the intervention group (green mussel shell) (U/L).

Green mussel	Mean ± SD	p	Levene
Week-2 ^nd^ (P _3_)	60.50 ± 34.88	0.052	0.034
Week-4 ^th^ (P _2_)	57.33 ± 14.72		
Week-6 ^th^ (P _1_)	18.65 ± 5.40		

Table 4 shows the results of the one-way ANOVA test reported a p-value = 0.510 and the Levene value = 0.772. Because the p-value was > 0.05, it can be concluded that the serum ALP levels in the negative control group (K _1.3_, K _1.2_, and K _1.1_) were not significantly different.

**Table 4.** One-way ANOVA test results for serum alkaline phosphatase levels in rabbits in the negative control group (U/L).

Control	Mean ± SD	p	Levene
Week-2 ^nd^ (K _1.3_)	72.00 ± 11.47	0.510	0.772
Week-4 ^th^ (K _1.2_)	57.70 ± 17.23		
Week-6 ^th^ (K _1.4_)	62.53 ± 16.02		

Table 5 shows that from the one-way ANOVA test results, the p-value = 0.416 and the Levene value = 0.192. Because the p-value was > 0.05, it can be concluded that the serum ALP levels in the week 2 were not significantly different.

**Table 5.** The results of the one-way ANOVA test for serum alkaline phosphatase levels of positive control, treatment, and negative control groups at week-2 ^nd^ (U/L).

Week-2 ^nd^	Mean ± SD	p	Levene
Positive control (bovine) (K _2.3_)	44.33 ± 19.00	0.416	0.192
Intervention (green mussel) (P _3_)	60.50 ± 34.88		
Negative control (K _1.3_)	72.00 ± 11.47		

Table 6 shows that the results of the one-way ANOVA test reported a p-value = 0.959 and the Levene value = 0.454. Because the p-value was > 0.05, it can be concluded that the serum ALP levels in the week 4 were not significantly different.

**Table 6.** The results of the one-way ANOVA test for serum alkaline phosphatase levels of positive control, treatment, and negative control groups at week-4 ^th^ (U/L).

Week-4 ^th^	Mean ± SD	p	Levene
Positive control (bovine) (K _2.2_)	60.40 ± 7.24	0.959	0.454
Intervention (green mussel) (P _2_)	57.33 ± 14.72		
Negative control (K _1.2_)	57.70 ± 17.23		

Table 7 shows that from the one-way ANOVA test results, the p-value = 0.030 and the Levene value = 0.087. Because the p-value was < 0.05 and a Levene value > 0.05, it can be concluded that the serum levels of ALP in the positive control, treatment, and negative control groups at week 6 had significant differences and were homogeneous. The test was continued with the LSD post hoc test to find out the differences between the treatment groups.

**Table 7.** The results of the one-way ANOVA test for serum alkaline phosphatase levels of positive control, treatment, and negative control groups at week-6 ^th^ (U/L).

Week 6 ^th^	Mean ± SD	p	Levene
Positive control (bovine) (K _2.1_)	42.17 ± 25.91	0.030	0.087
Intervention (green mussel) (P _1_)	18.65 ± 5.40		
Negative control (K _1.1_)	62.53 ± 16.02		

Table 8 above shows that the results of the post hoc LSD test reported the positive control group (bovine) (K _2.1_) against the treatment group (green mussel shells) (P _1_) and the negative control group (K _1.1_) were not significantly different, while the treatment group (green mussel shells) (P _1_) against the negative control group (K _1.1_) had significant results.

**Table 8.** The results of the LSD post hoc test for serum alkaline phosphatase levels of positive control, treatment, and negative control groups at week-6 ^th^ (U/L).

Group		p	Description
I	II		
Positive control (bovine) (K _2.1_)	Intervention (green mussel) (P _1_)	0.107	Not significant
	Negative control (K _1.1_)	0.178	Not significant
Intervention (green mussel) (P _1_)	Negative control (K _1.1_)	0.011	Significant

**Figure 2.**
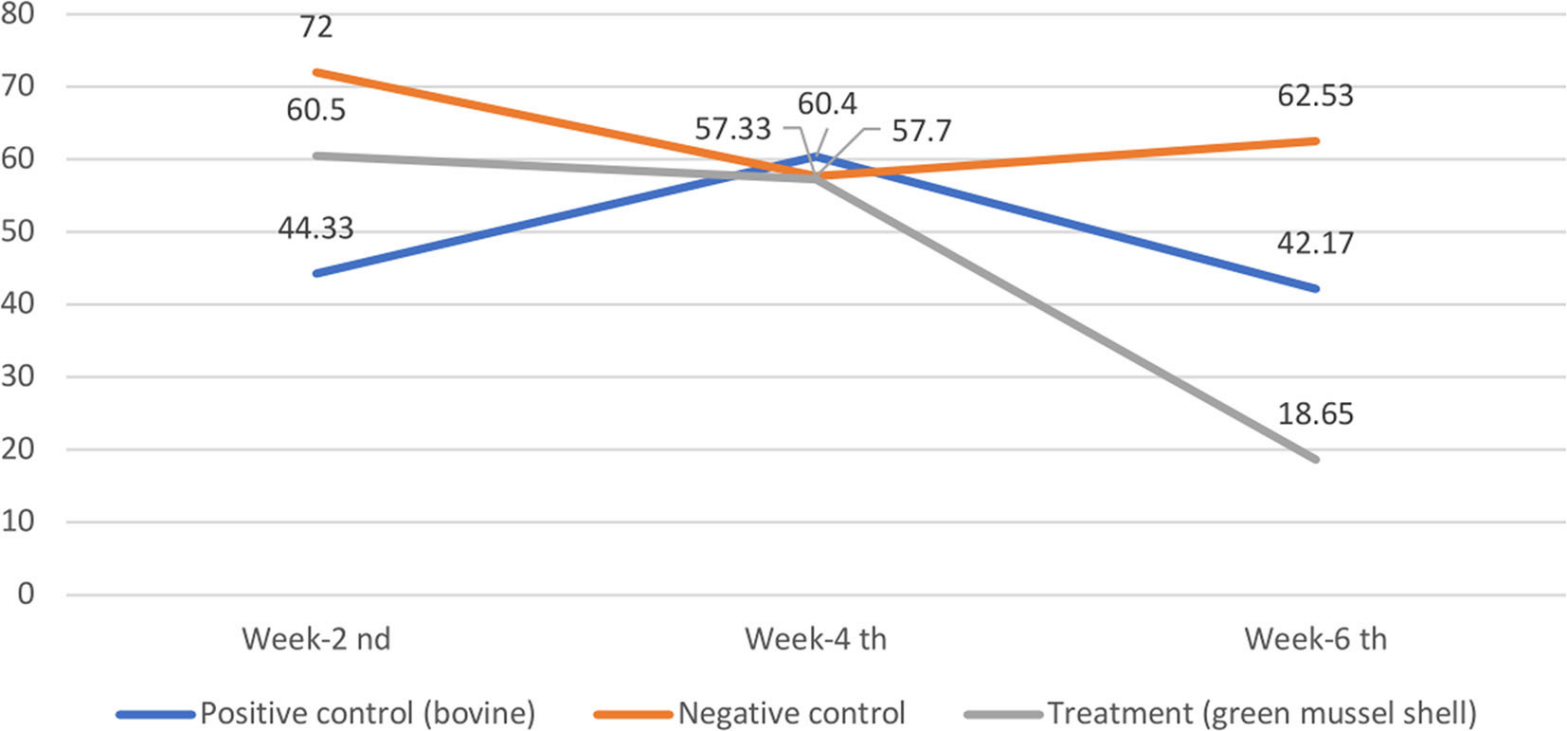
Graph Rabbit serum alkaline phosphatase level (U/L).

Based on Tables 1- 8 in that study, a comparison was made between the characteristics of hydroxyapatite (HA) from green mussel shells and bovine HA. The characteristics of green mussel shell HA included the content of calcium oxide (CaO), phosphorus pentoxide (P _2_O _5_), and molecular weight, which differed from bovine HA. Furthermore, cytotoxic tests for both types of HA yielded results supporting their safe use in medical applications.

In vivo evaluations such as radiology and histology also supported the findings regarding the effectiveness of green mussel shell HA in accelerating bone healing. Radiology results showed increased bone density and better structure in areas treated with green mussel shell HA compared to control areas. Additionally, histology analysis showed better bone regeneration and a positive tissue response in areas treated with green mussel shell HA, confirming the ability of this material to enhance the bone healing process. Thus, the findings from the evaluation of HA characteristics, cytotoxic assays, and in vivo radiology and histology results provided strong support for the effectiveness and safety of using green mussel shell HA in accelerating bone healing and demonstrated the potential of this material as a promising alternative in clinical applications.

## Discussion

The osteoconductive matrix is one of the elements that influences the bone healing process. It serves as a scaffold or mineralizing agent for bone and helps in the adherence of osteoconductive and osteogenic cells to the fracture site. Hydroxyapatite is one form of an osteoconductive matrix (HA). Hydroxyapatite is a substance made up of calcium (Ca ^2+^), hydroxy (OH-), and phosphate (PO _4_^3-^) ions that form a crystal structure with the chemical formula Ca _5_(PO _4_) _3_(OH). The body will naturally generate hydroxyapatite from Ca ^2+^ ions in the blood and inorganic phosphate compounds (P _i_ or PO _4_^3-^). Compound P _i_ is generated by dephosphorylating organic phosphate compounds (PP _i_), which are phosphates that happen at the ALP dephosphorylation process. As a result, the presence of a hydroxyapatite scaffold reduces ALP activity since the body no longer needs to synthesize hydroxyapatite from PP _i_^29^ refers to the localized effect in bone tissue. When hydroxyapatite (HA) is applied as a scaffold in bone tissue, it provides the necessary mineral components directly, reducing the body's need to produce HA from inorganic phosphate compounds. This reduction in synthesis activity may lead to lower serum ALP levels, reflecting a decreased demand for bone mineralization processes.

Because of that, ALP levels in a bone healing union will be normal or slightly elevated since the hydroxyapatite in green mussel shells functions well as a scaffold. In contrast to non-union fractures, the bone healing process takes longer, and more phosphate is required to compensate for the failure of bone healing, causing ALP levels to grow higher than in union fractures. This condition serves as the study’s measurement basis. ^22^ However, ALP does not only play a role in the bone healing process, but also plays a role in other organ systems, such as hepatobiliary, genitourinary, and gastrointestinal. ^30^ As a result, it is important to ensure that these body functions are in good condition so that false negatives or false positives do not occur.

This study aims to determine the effect of green mussel shell hydroxyapatite on blood alkaline phosphatase (ALP) levels in bone grafting procedures in New Zealand rabbits. The total sample was 36 New Zealand rabbits which were then divided into 9 groups. Following this, observations were made in the groups of negative control, positive control groups, and intervention groups in the weeks 2, 4, and 6. Using a surgical procedure and drilling with a width and depth of 5 mm, all groups produced lesions on the femoral diaphysis. The treatment group received green mussel shell hydroxyapatite (HA), while the negative control group did not get any HA as a follow-up intervention. The positive control group received bovine hydroxyapatite (HA). Drill lesions were made to develop the femoral diaphysis to replicate a fracture and to provide a suitable area to apply HA to the cavity formed by the drilling process. In this experiment, an effective dose of bone healing was up to 300 mg of the bone graft. ^12^

This experiment had a number of methodological limitations, including fractures in the rabbits and post-interventional infections that were not equally exposed to all of the rabbits. This may impact the trial outcomes by increasing the time it takes for bones to recover, which could prevent all treatment groups from showing significant results between the weeks 2, 4, and 6. Even though all groups received an appropriate dose of 300 mg at the area of the lesion, the prolonged bone healing time meant that the test between the positive control group and the negative control group did not produce significant results in the week 6.

According to data analysis, the only test that produced significant results with a p-value less than 0.05 on the post-hoc LSD test was the comparison between the week 6 negative control group (K _1.1_) and the week 6 treatment group (P _1_). The presence of blood clots in bone defects in the negative control group will initiate the natural bone healing cascade, leading to the organization of blood clots and subsequent bone regeneration. This natural process is crucial for bone healing and can result in the formation of new bone over time. Significant differences between the negative control group and the treatment group may indicate that the intervention of green mussel shell hydroxyapatite does not show superior effectiveness compared to natural blood clots in promoting bone healing in this experimental setting. This demonstrates the effectiveness of green mussel shell HA in accelerating and maximizing the healing of bone fractures. However, it could not detect a significant difference between the week 2 and 4. This shows that the week 6, when ALP activity is at its peak, is the optimal week to observe how HA treatment affects the bone-healing process in femoral fractures as compared to weeks 2 and 4. ^31^

Additionally, even though there were no statistically significant differences between the week 6 positive control group (K _2.1_) and the week 6 negative control group (K _1.1_) in the test, K _2.1_’s ALP level was lower than K _1_’s. This demonstrates that bovine HA can speed up bone repair, but its effectiveness is lower than that of HA from green mussel shells. The potential allergic reactivity of hydroxyapatite (HA) originating from green mussel shells in the human body is deemed to be minimal. This is due to the fact that shellfish allergens typically consist of proteins, rather than mineral components such as HA. The synthesis process of HA from green mussel shells entails extensive purification to eliminate organic substances, including proteins, which serve as the primary allergens. Nonetheless, it is of utmost importance to ensure that the purification process is conducted thoroughly to eradicate any residual proteins that may induce allergic responses. Further investigation and clinical trials are imperative to ascertain the biocompatibility and safety of this substance in humans.

A comparison test of the serum ALP levels at the weeks 2, 4, and 6 in each treatment group’s results did not reveal any significant differences. This indicates that while ALP activity varied at weeks 2, 4, and 6, the differences were not statistically significant. This means that the effect of giving HA works evenly in the weeks 2, 4, and 6. This was done because the main focus of the study was probably on the evaluation of serum alkaline phosphatase (ALP) levels as a biochemical marker of bone healing response to hydroxyapatite treatment. The researchers thus prioritized this aspect of the investigation over histological analysis.

## Conclusion

This study investigates the effects of green mussel shell hydroxyapatite (HA) on bone healing in rabbits, comparing it with bovine HA and a negative control. The findings demonstrate that green mussel shell HA significantly enhances bone healing, particularly evident at week 6, where there is a notable difference in serum alkaline phosphatase (ALP) levels compared to the negative control group. This indicates that green mussel shell HA not only accelerates the bone healing process but also improves the overall quality of bone repair more effectively than bovine HA. Green mussel hydroxyapatite (HA) is proven able to fasten and maximize the bone healing process as fast as bovine HA and even has higher efficacy than bovine HA. In summary, green mussel shell hydroxyapatite (HA) contributes to the bone healing process by providing a bioactive scaffold that enhances cellular response, promotes osteoblast differentiation, and supports mineralization. Although the natural healing process over time is a significant factor, the use of green mussel shell HA appears to expedite and improve the overall quality of bone repair. This study highlights the potential of green mussel shell HA as a superior bone substitute, offering a promising avenue for future research and clinical applications.

## Data Availability

Mendeley Data: Green Mussel Shell Hydroxyapatite ALP Data Set, https://data.mendeley.com/datasets/gvhpb34kkr/1. ^32^ Data are available under the terms of the Creative Commons Attribution 4.0 International license (CC-BY 4.0). ARRIVE checklist for ‘The effect of green mussel (Perna viridis) shells’ hydroxyapatite application on alkaline phosphatase levels in rabbit femur bone defect’, https://doi.org/10.17632/vyw756k44g.1. Data are available under the terms of the Creative Commons Attribution 4.0 International license (CC-BY 4.0).
