# Supplementary material for: The effect of green mussel ( Perna viridis) shells’ hydroxyapatite application on alkaline phosphatase levels in rabbit femur bone defect
Source: F1000Res. 2024 Jun 7;12:631. Originally published 2023 Jun 8. [Version 2] doi: 10.12688/f1000research.132881.2 (PMC11195609; doi:10.12688/f1000research.132881.2)
Supplement: Peer review history

# Reviewer report: Reviewer response for version 2

**DOI:** 10.5256/f1000research.166847.r288125

**Published:** 2024-06-19

**Authors:** Happy Kurnia Permatasari, Fahrul Nurkolis, Rudy Kurniawan

**Affiliations:** 1 Department of Biochemistry and Biomolecular, Faculty of Medicine, Brawijaya University, Malang, Indonesia; 2 Biological Sciences, State Islamic University of Sunan Kalijaga (UIN Sunan Kalijaga Yogyakarta), Yogyakarta, Yogyakarta, 55281, Indonesia; 3 Graduate School of Medicine, Hasanuddin University (Ringgold ID: 64739), Makassar, South Sulawesi, Indonesia

The authors have made significant changes to the manuscript, which has positively affected its quality.

Is the work clearly and accurately presented and does it cite the current literature?

Partly

If applicable, is the statistical analysis and its interpretation appropriate?

Partly

Are all the source data underlying the results available to ensure full reproducibility?

Yes

Is the study design appropriate and is the work technically sound?

Partly

Are the conclusions drawn adequately supported by the results?

Partly

Are sufficient details of methods and analysis provided to allow replication by others?

No

Reviewer Expertise:

In silico, in-vitro and in-vivo studies; natural biomaterials; nutraceuticals; functional food; biochemistry and biomolecular; natural product

We confirm that we have read this submission and believe that we have an appropriate level of expertise to confirm that it is of an acceptable scientific standard.

# Reviewer report: Reviewer response for version 2

**DOI:** 10.5256/f1000research.166847.r288127

**Published:** 2024-06-18

**Authors:** George J Dias

**Affiliations:** 1 University of Otago, Dunedin, New Zealand

I am happy with the revisions made by the authors. I consider that this manuscript is ready to be Indexed.

Is the work clearly and accurately presented and does it cite the current literature?

Partly

If applicable, is the statistical analysis and its interpretation appropriate?

Yes

Are all the source data underlying the results available to ensure full reproducibility?

Yes

Is the study design appropriate and is the work technically sound?

Partly

Are the conclusions drawn adequately supported by the results?

Partly

Are sufficient details of methods and analysis provided to allow replication by others?

Partly

Reviewer Expertise:

Biomaterials, in-vitro and in-vovo investigations.Implants for surgical applications.

I confirm that I have read this submission and believe that I have an appropriate level of expertise to confirm that it is of an acceptable scientific standard.

# Reviewer report: Reviewer response for version 1

**DOI:** 10.5256/f1000research.145833.r200155

**Published:** 2023-09-06

**Authors:** Maria Apriliani Gani

**Affiliations:** 1 Pharmacology-Clinical Pharmacy, Bandung Institute of Technology, Bandung, West Java, Indonesia; 2 Universitas Airlangga, Surabaya, East Java, Indonesia

The manuscript “The effect of green mussel (Perna viridis) shells’ hydroxyapatite application on alkaline phosphatase levels in rabbit femur bone defect” was aimed to observe the effectiveness of green mussel shell HA as a bone substitute material.

 Before being indexed, the article must go through a major revision, including data addition and extensive English editing.

**Abstract:**

- It is recommended to summarize the background section in the abstract and detail the aim of the study.
- In the method section of the abstract, kindly briefly mention the kind of evaluation that was used in the study (histology, etc.).
- The results section in the abstract should be written in the past tense.

**Introduction:**

- Kindly check and revise the grammar in this section. Please be aware of when to use past tense etc.
- “If these conditions are not handled, the decline in quality of life and activity limitations cannot be avoided.” Kindly add the data based on another study regarding quality of life and fracture incidence rate before this sentence.
- “As a result, bone grafts with osteogenesis, osteoinduction, and osteoconduction characteristics are required to assist in the healing of acute fractures and non-union fractures.” Please add the reference for this.
- “A substance called hydroxyapatite (HA)”. I recommend the authors to change the word “substance” to “material.”
- “Virgin clam shells and green mussel shells are just two examples of wastes that could serve as a source of HA for bone substitutes.” Kindly delete the word “just.”
- The author should explain why virgin clam shells and green mussel shells are recommended to be used as HA sources. What are the differences between them with other natural sources of HA?
- The author should detail the hypothesis of the use of green mussel shells in the bone healing process, in terms of the chemical and physical characteristics of this material.
- “Alkaline phosphatase (ALP), an enzyme that contributes to the process of bone mineralization, is produced by osteoblasts with increased activity in the third stage.” Delete the “third stage” or keep it with prior explaining about bone healing stages.
- “As one of the elements of a complete blood count, ALP can therefore serve as a biomarker that is frequently evaluated and easily accessed to detect bone healing.” The authors should detail the correlation of bone healing processes with ALP serum levels not only in humans but also in animal models. Does it really have a positive correlation of not statistically differ based on other papers?

**Methods:**

- I recommend the authors summarize the methods section.
- “The inclusion criteria in this study were skeletally matured, male New Zealand rabbits (Oryctolagus cuniculus), aged 6-12 months, with a weight of 2.5 – 3 kg. The exclusion criteria in this study were there being anatomical abnormalities, signs of infection, and rabbits that died during treatment.” The inclusion and exclusion criteria mean that any criteria chosen before the study begins. Rabbits who died during the treatments are not included in the criteria. Any animals that died during the treatment must be noted, observed, and reported in the manuscript.
- There was a high number of deaths of the animal and it is a must that the authors should clearly explain the cause of death in the manuscript.
- Does the defect model present the “non-union fractures”? The authors should mention the criteria of non-union fracture and match it with the defect model that was created in the study.
- The authors treated the rabbit with HAs in powder form. How do the authors ensure that the material can serve as a scaffold in the defective tissue? Since HAs may combine with the blood in the defect site it makes the “scaffold” and the treatment fail.
- Please explain why the authors use bovine HA as the positive control, as well as the company of the bovine HA that was used in this study.
- Table 1 is not needed to be included in the manuscript, it is enough to mention and cite the source.
- The authors should detail the product code and company that provided the ALP kit.

**Results:**

- Table 2-9 came from the same data set, which was the ALP levels. These tables can be present in one graph only.
- The authors must add another data set in the manuscript, including:

1. Characteristics of green mussel shells HA and bovine HA.
2. Cytotoxic test for the HAs.
3. Other in vivo evaluation results such as radiology and histology to support the current findings.

**Discussion:**

- The authors should detail the concept of ALP levels in each timeline in the bone healing process. When usually the ALP reaches its peaks etc. and why.

- “As a result, the presence of a hydroxyapatite scaffold reduces ALP activity since the body no longer needs to synthesize hydroxyapatite from PPi.” This process occurs in the bone tissue. The authors should include the correlation of the tissue and serum ALP levels.
- Since the HA came from shells, is there any potential allergic reactivity of the developed material in the human body?

**References:**

- Please update the references with the up-to-date articles.

Is the work clearly and accurately presented and does it cite the current literature?

Partly

If applicable, is the statistical analysis and its interpretation appropriate?

Yes

Are all the source data underlying the results available to ensure full reproducibility?

Yes

Is the study design appropriate and is the work technically sound?

Partly

Are the conclusions drawn adequately supported by the results?

Yes

Are sufficient details of methods and analysis provided to allow replication by others?

Yes

Reviewer Expertise:

Biomaterials, biomedical pharmacy, pharmacology.

I confirm that I have read this submission and believe that I have an appropriate level of expertise to confirm that it is of an acceptable scientific standard, however I have significant reservations, as outlined above.

# Reviewer report: Reviewer response for version 1

**DOI:** 10.5256/f1000research.145833.r200163

**Published:** 2023-09-06

**Authors:** Happy Kurnia Permatasari, Fahrul Nurkolis, Rudy Kurniawan

**Affiliations:** 1 Department of Biochemistry and Biomolecular, Faculty of Medicine, Brawijaya University, Malang, Indonesia; 2 Biological Sciences, State Islamic University of Sunan Kalijaga (UIN Sunan Kalijaga Yogyakarta), Yogyakarta, Yogyakarta, 55281, Indonesia; 3 Graduate School of Medicine, Hasanuddin University (Ringgold ID: 64739), Makassar, South Sulawesi, Indonesia

We have fully and carefully evaluated this manuscript, "The effect of green mussel ( *Perna viridis*) shells' hydroxyapatite application on alkaline phosphatase levels in rabbit femur bone defect" by Tjandra *et al*.

 Here are our comments:

 1. First of all, we agree with the other reviewer who requires the authors to re-edit the abstract section. The aims of this research are not yet visible in the abstract. This "better than HA bovine" refers to control? Or which intervention? Because it is not mentioned in the abstract methods, but appears in the results.

2. Introduction:

- Provide the latest data and a wider area, it is stated that the authors adapted data from "Regional Police of Central Java in 2018". This is a small part of Indonesia and does not reflect the urgency in the world; and some data got "2008 to 2009", please fix it to be more updated.
- The aim of the study must also be addressed from the perspective of describing the contribution to the field under analysis and the elements of scientific novelty presented, as the **last, separate** paragraph of this section, to make it more easily visible. Develop it better. What differentiates your paper from others on the same topic? Give a reason for interest in this paper.

3. Methods:

- Register this research protocol at https://preclinicaltrials.eu and mention the registration number in the methods section; it can be registered at any time and for free, this is to increase the readability value of this article.
- Writing the Latin name ***Oryctolagus cuniculus*** at the beginning can be long, but from now on it must be *O. cuniculus*; and just write the Latin name without " **Rabbits**"; to be more efficient.
- Analysis: Data analysis is processed using what software? With what CI? It should be clear and detailed.
- How is the sample size calculated? What formula do the authors use?
- What is the HA dosage given based on?

4. Results:

 It would be more valid to display tissue histology figure observation data from Animal Experimental to prove the authors' claim to the conclusion that *"Green mussel hydroxyapatite (HA) is proven able to fasten and maximize the bone healing process as fast as bovine HA and even has higher efficacy than bovine HA.*"

5. Discussion:

 A possible mechanism for the bone healing process must be discussed in a comprehensive manner, due to the long research, this raises the question of whether Green mussel hydroxyapatite (HA) really contributes to the healing process or if it is due to the healing process over time.

 6. Very brief conclusion, elaborate it with the findings that have been observed in the research.

 We conclude that this article needs to be extensively revised.

Is the work clearly and accurately presented and does it cite the current literature?

Partly

If applicable, is the statistical analysis and its interpretation appropriate?

Partly

Are all the source data underlying the results available to ensure full reproducibility?

Yes

Is the study design appropriate and is the work technically sound?

Partly

Are the conclusions drawn adequately supported by the results?

Partly

Are sufficient details of methods and analysis provided to allow replication by others?

No

Reviewer Expertise:

In silico, in-vitro and in-vivo studies; natural biomaterials; nutraceuticals; functional food; biochemistry and biomolecular; natural product

We confirm that we have read this submission and believe that we have an appropriate level of expertise to confirm that it is of an acceptable scientific standard, however we have significant reservations, as outlined above.

# Reviewer report: Reviewer response for version 1

**DOI:** 10.5256/f1000research.145833.r195140

**Published:** 2023-08-24

**Authors:** George J Dias

**Affiliations:** 1 University of Otago, Dunedin, New Zealand

The Abstract needs to be improved.

***Page 5**: Seven rabbits from seven different groups (P* *1* *, K* *1.1* *, P* *2* *, K* *2.2* *, P* *3* *, K* *2.3* *, K* *1.3* *) were found dead during daily monitoring several days after the surgery with no specific cause of death.*

 This is a relatively high number of deaths, 7 out of 36 - ~20%. Very surprising that cause of death such as infection, haemorrhage was not determined?

*..., UV* *sterilization for* *3 hours was conducted.*

 I consider this sterilization method to be inadequate for a material to be surgically implanted. Because UV light will only disinfect the surface and will not have an effect of the interior of the implant particles. Authors need to provide evidence about how this sterilization method was selected using general surgical principals.

**Page 6**: ... *using a drill.*

 Give more details of the bone drilling process. What was the speed of the motor? The type and size of the bur? Was the bur irrigated during drilling?

*fill the defect with green mussel shell HA.*

 What was the form of HA? Was it a powder? If so what is the size of the particles?

*The blood sampling was conducted by inserting needle parallel to the lateral marginal auricular vein. Once the needle* *reached the vein, blood will flow into the catheter. The vacutainer tube was pushed so that blood can enter the 1-2 ml tube.* *After collecting the required sample, the needle was immediately removed from the rabbit* *’* *s lateral marginal vein. The injected area was pressed with an alcohol swab at the end of this process.*

 It is not necessary to provide minute details on venopuncture. It would have been preferable if this much of details were provided for the surgical technique of HA implantation.

*Examination of ALP is selected due to its availability in most health centers.*

 Question is blood samples tested in Animal Health Centres are that of common companion animals such as dogs and cats. Therefore, can testing ALP in rabbit blood in a health centre be accurate?

**Page 9:** *As a result, the presence of a hydroxyapatite scaffold* *reduces ALP activity since the body no longer needs to synthesize hydroxyapatite from PPi.*

 It can also be argued that the low ALP level indicate that the rate of bone remodelling is slow, leading to the rate of new bone formation to be also being low.

*This experiment had a number of methodological limitations, including fractures in the rabbits and post-interventional* *infections that were not equally exposed to all of the rabbits. This may impact the trial outcomes by increasing the time it* *takes for bones to recover, which could prevent all treatment groups from showing significant results between the weeks* *2, 4, and 6. Even though all groups received an appropriate dose of 300 mg at the area of the lesion, the prolonged bone*

*healing time meant that the test between the positive control group and the negative control group did not produce* *significant results in the week 6.*

 If most of the animals in this study experienced delayed healing of surgical sites due to infections, then the outcome of the complete investigation is brought into question?

*..... negative control group (K* *1.1)*

 In the negative control, although there was no implant material in the surgical site, the bone defect would not have been empty, it would have been filled with blood clot that eventually got organised, leading to bone healing with new bone formation. Therefore, the significant difference between negative group and the treatment group may indicate that the green mussel shell hydroxyapatite may not be effective as the natural blood clot?

*A comparison test of the serum ALP levels at the weeks 2, 4, and 6 in each treatment group* *’* *s results did not reveal any* *significant differences. This indicates that while ALP activity varied at weeks 2, 4, and 6, the differences were not statistically significant. This means that the effect of giving HA works evenly in the weeks 2, 4, and 6.*

 It is very curious why the authors did not take bone tissue biopsies from the surgical sites at these time points and investigate the histology, and compare these findings with that of the ALP values?

Is the work clearly and accurately presented and does it cite the current literature?

Partly

If applicable, is the statistical analysis and its interpretation appropriate?

Yes

Are all the source data underlying the results available to ensure full reproducibility?

Yes

Is the study design appropriate and is the work technically sound?

Partly

Are the conclusions drawn adequately supported by the results?

Partly

Are sufficient details of methods and analysis provided to allow replication by others?

Partly

Reviewer Expertise:

Biomaterials, in-vitro and in-vovo investigations.Implants for surgical applications.

I confirm that I have read this submission and believe that I have an appropriate level of expertise to state that I do not consider it to be of an acceptable scientific standard, for reasons outlined above.
